# Rapid Antibiotic Resistance Serial Prediction in *Staphylococcus aureus* Based on Large-Scale MALDI-TOF Data by Applying XGBoost in Multi-Label Learning

**DOI:** 10.3389/fmicb.2022.853775

**Published:** 2022-04-12

**Authors:** Jiahong Zhang, Zhuo Wang, Hsin-Yao Wang, Chia-Ru Chung, Jorng-Tzong Horng, Jang-Jih Lu, Tzong-Yi Lee

**Affiliations:** ^1^Warshel Institute for Computational Biology, The Chinese University of Hong Kong, Shenzhen, China; ^2^School of Life and Health Sciences, The Chinese University of Hong Kong, Shenzhen, China; ^3^School of Life Sciences, University of Science and Technology of China, Hefei, China; ^4^Department of Laboratory Medicine, Chang Gung Memorial Hospital at Linkou, Taoyuan, Taiwan; ^5^Ph.D. Program in Biomedical Engineering, Chang Gung University, Taoyuan, Taiwan; ^6^Department of Computer Science and Information Engineering, National Central University, Taoyuan, Taiwan; ^7^Department of Bioinformatics and Medical Engineering, Asia University, Taichung, Taiwan; ^8^Department of Medical Biotechnology and Laboratory Science, Chang Gung University, Taoyuan, Taiwan; ^9^Department of Medicine, College of Medicine, Chang Gung University, Taoyuan, Taiwan

**Keywords:** MALDI-TOF MS, oxacillin resistance, clindamycin resistance, XGBoost, multi-label learning

## Abstract

Multidrug resistance has become a phenotype that commonly exists among *Staphylococcus aureus* and is a serious concern for infection treatment. Nowadays, to detect the antibiotic susceptibility, antibiotic testing is generated based on the level of genomic for cure decision consuming huge of time and labor, while matrix-assisted laser desorption-ionization (MALDI) time-of-flight mass spectrometry (TOF/MS) shows its possibility in high-speed and effective detection on the level of proteomic. In this study, on the basis of MALDI-TOF spectra data of discovery cohort with 26,852 samples and replication cohort with 4,963 samples from Taiwan area and their corresponding susceptibilities to oxacillin and clindamycin, a multi-label prediction model against double resistance using Lowest Power set ensemble with XGBoost is constructed for rapid susceptibility prediction. With the output of serial susceptibility prediction, the model performance can realize 77% of accuracy for the serial prediction, the area under the receiver characteristic curve of 0.93 for oxacillin susceptibility prediction, and the area under the receiver characteristic curve of 0.89 for clindamycin susceptibility prediction. The generated multi-label prediction model provides serial antibiotic resistance, such as the susceptibilities of oxacillin and clindamycin in this study, for *S. aureus*-infected patients based on MALDI-TOF, which will provide guidance in antibiotic usage during the treatment taking the advantage of speed and efficiency.

## Introduction

The multidrug resistance phenotype that occurred within *Staphylococcus aureus* is considered as one of the most intractable pathogenic features in the history of antibiotic chemotherapy (Hiramatsu et al., 2014). This feature refers to *Staphylococcus aureus*, which shows resistance to a set of antibiotics. oxacillin-resistant *S. aureus* (ORSA) has been increasing in importance as a leading cause of both nosocomial and community-acquired infections (Bell and Turnidge, 2002). Similar to penicillin and methicillin, oxacillin belongs to β-lactam drugs. The initial discovery on the mechanism of β-lactam drugs is the existence of penicillin-binding-proteins (PBPs), which are transpeptidases responsible for partial peptidoglycan construction on cell walls. The binding between penicillin and PBPs blocks the function of PBPs and creates the entry for penicillin. Gene blaZ was induced in bacteria encoding a β-lactamase enzyme, which opens up the β-lactam ring at the core of penicillin, preventing the binding to PBPs. Oxacillin resistance results from a new PBP, decreasing the affinity for oxacillin, though the β-lactam ring within the drug has been modified and stabilized. Clindamycin-resistant *Staphylococcus aureus* (CRSA) is free of the suppression in the virulence factors expression, which is originally regulated by clindamycin (Hodille et al., 2018). The mechanism of clindamycin is binding to the ribosome and inhibiting protein synthesis (Kehrenberg et al., 2005). Correspondingly, clindamycin resistance results from conformation change of ribosome induced by enzymes, which leads to the affinity decreasing (Reygaert, 2013).

Nowadays, to test antibiotic susceptibility, the workflow takes 24–72 h including disk diffusion. Basically, in the case of the low-efficiency treatment, patients infected by ORSA or CRSA are asked to do the test and wait for the detection result (Swenson et al., 2001), which causes a delay for the concise and precise treatment individually ranging from 24 to 72 h, though broad-spectrum empirical treatment would be conducted. Besides, long-time testing is not suitable for urgent patients and leaves the time lag for the probability of mutation.

In recent years, a huge number of arrays or kits emerged and be applied in clinical detection such as Velogene and MRSA-Screen, improving the detection time within 4 h (Louie et al., 2000). For instance, Velogene uses a chimeric probe aiming at the mecA gene within 90 min. Nevertheless, the high cost of detection kits and limited labor capacity, privacy policy restricts the application of genome detection. Proteomic of the resistant *S. aureus* is also a focus of identification based on the ion types and expression intensity generated by the spectra. Current antibiotic susceptibility tests have shortened the detection time within several hours besides *S. aureus* isolation and culture. Nevertheless, the time lag still exists the chance for resistance induction, which is calling for rapid detection and proteomic-based tests with statistics and computational algorithms. Specifically, with the availability of matrix-assisted laser desorption-ionization (MALDI)-time-of-flight mass spectrometry (TOF/MS), its fast generation speed and accurate fragmentation detection are the advantages as well as cross-species processing, which are longed for a long time to solve resistance detection.

Matrix-assisted laser desorption-ionization-time-of-flight mass spectrometry is a special kind of mass spectrometry technique that requires protein samples crystallized within the matrix for further ionization and detection, which can be applied to grasp the resistant characters besides antibiotic susceptibility testing (Lay, 2001; Croxatto et al., 2012). Each run of detection through MALDI-TOF only causes low cost within a few dollars within 5 min. Scientists have tried to combine the statistical analysis, computational method, even machine learning with the spectra information such as mass-to-charge (*m*/*z*) ratio and peptide intensity from the MALDI-TOF to differentiate sensitive and resistant *S. aureus* for several types of antibiotics (Wang et al., 2020). Through the combination of MALDI-TOF and machine learning, the classification model could be a guide to provide insight information into drug susceptibility during the clinical treatment and even show the potential of saving the antibiotic test in the ideal case.

The crucial consideration from both patients and doctors is that the computational model on the basis of the cohort representation and assumption lacks quality guarantee for individuals, which can be solved and ensured largely in the antibiotics susceptibility test. Specifically, the consideration is getting mitigated with a novel resistance information database called DRIAMS with huge-scale data, which collects at least 300,000 mass spectra with more than 750,000 antimicrobial resistances (Weis et al., 2022). Another limitation is that each existing classification model only refers to a specific type of antibiotic, which is not suitable and applicable for the multidrug-resistant *S. aureus* with the widespread multidrug-resistant phenotype, referring to being resistant to at least three classes of antibiotic mechanisms or three antibiotics based on the gene level (Schwarz et al., 2010). Thereby, to relieve the dilemma in cohort representation and size, this study recruited 26,852 patients infected by *S. aureus* in the Chang Gung Memorial Hospitals (CGMH) at the Linkou branch from 2013 to 2019. Antibiotic susceptibility tests on oxacillin and clindamycin had been conducted for the samples, and their *S. aureus* susceptibility status was mapped with their MALDI-TOF results as the labels. Besides, for the reproducibility, from 2015 to 2017, this study also recruited 4,963 patients as the validation test for the constructed model at the Kaohsiung branch. In our dataset, information, such as specimen type, sex, age, *m*/*z*, and peak intensity, is included for each sample. Two drug susceptibilities are combined in the form of a tuple as the label data. We aimed to construct a prediction model using the high-dimension and large-scale MALDI-TOF data to indicate the resistance for oxacillin and clindamycin in patients, which is not the typical case for multiresistance but breaking model mode for single susceptibility prediction. Meantime, this study applies the XGBoost algorithm in multi-label learning for fast and accurate serial resistance prediction. Over the long haul, the model could improve resistance detection, provide medication guidance, and be extended to serial antibiotic susceptibility tests.

## Materials and Methods

### Overview of the Study

**FIGURE 1:**
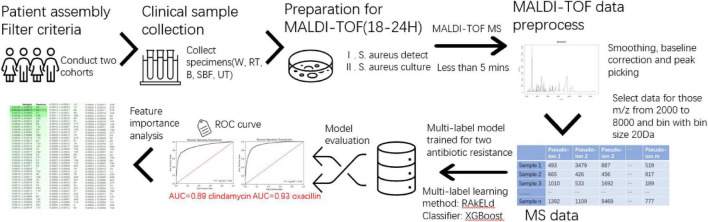
Overview of the study. The flowchart mainly contains sample collection, matrix-assisted laser desorption-ionization-time-of-flight mass spectrometry (MALDI-TOF) process, and multi-label model construction and evaluation.

This study consists of sample data generation and prediction model construction (as shown in Figure 1). During the first step, the clinical specimen from recruited patients is cultured, and a single pathogen colony after isolation and incubation was treated with the MALDI-TOF MS spectra. During the second step, the mass spectra data generated by the MALDI-TOF from different samples went through the preprocessing and were used for the modeling of serial-drug resistance prediction. Figure 1 presents the overlook of this study including the basic flow of the MALDI-TOF MS spectra and the process of model construction. Study details are introduced in the following sections.

### Experiment Cohorts’ Information, MALDI-TOF Preparation, and Processing

Two cohorts, Linkou and Kaohsiung, are used as the discovery and replication population, respectively, which is independent of each cohort. The Linkou cohort focused on the oxacillin and clindamycin resistance of *S. aureus* lasting from 2013 to 2019. We collected wound (W) swab specimens, respiratory tract (RT), sterile body fluid (SBF), blood (B), and urinary tract (UT) from patients from different departments during the data tracking. For those samples that showed resistance in the AST, CGMH cultured the clinical specimens, isolated bacterial pathogens from the samples, and did the antibiotic resistance profiling. Table 1 presents the label information of the Linkou cohort, which is prepared for the discovery part (Table 2). Notably, 26,852 samples in total are marked with two labels after the antibiotic susceptibility testing to the oxacillin and clindamycin. One of the labels can be categorized as ORSA or oxacillin-sensitive *Staphylococcus aureus* (OSSA). The other one can be presented as CRSA or clindamycin-sensitive *Staphylococcus aureus* (CSSA). Meanwhile, more information, such as age and sex, was collected from each participant. In the Kaohsiung cohorts (Table 2), 4,963 samples were collected from 2015 to 2017 as another independent cohort and treated with the consistent processing procedures as the Linkou cohort.

**TABLE 1:** Susceptibility information for clindamycin and oxacillin in the Linkou cohort.

Clindamycin\Oxacillin	Susceptible	Resistant	Total
Susceptible	11,453	3,761	15,214
Resistant	1,539	10,099	11,638
Total	12,992	13,860	26,852

**TABLE 2:** Susceptibility information for clindamycin and oxacillin in the Kaohsiung cohort.

Clindamycin\Oxacillin	Susceptible	Resistant	Total
Susceptible	2,303	800	3,103
Resistant	288	1,572	1,860
Total	2,591	2,372	4,963

**FIGURE 2:**
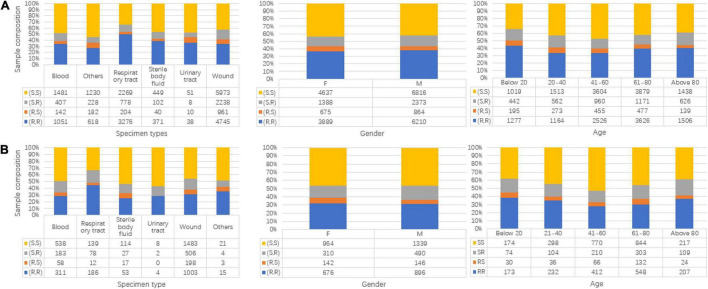
Data composition information in isolated *Staphylococcus aureus* for Linkou cohort in subgraph **(A)** (discovery population) and Kaohsiung cohort in **(B)** (replication population). The turtle in the graph stands for clindamycin susceptibility and oxacillin susceptibility. R is for resistant, and S is for susceptible. **(A)** Specimen types, gender, and age map to multi-labels in the discovery population. **(B)** Specimen types, gender, and age map to multi-labels in replication population.

Besides the label information, more basic information for samples including specimen types, gender, and age is shown in Figure 2. In each subgraph, the sample composition under each category is presented. For Figures 2A,B, the subgraphs in both mainly share the same composition situation corresponding to the same horizontal coordinate (specimen types, gender, and age).

Specimens are treated individually and separately with corresponding methods for sample culture. Notably, 1.2 ml of 0.9% saline solution is added to rinse the W swab specimens. Following, transfer equivalently onto four kinds of culture media including blood plate agar, eosin methylene blue agar, Columbia nalidixic acid, and chocolate agar. As for blood, we used a blood culture kit (BD BACTEC™ FX), which is for commercial use and from Becton, Dickinson and Company, to isolate pathogens. Following the positive blood culture bottle, we inoculated it on blood plate agar to regain single colonies. Sharing a similar protocol, such as W swab specimens, sterile body fluid is added onto the four agars, same as the W swab specimens, and rinsed by liquid thioglycolate for microorganisms’ isolation. After getting the culture prepared, agars and media were put into a 37°C CO_2_ incubator for 18–24 h. After the culture, we selected single colonies on the agar plate for the analysis of MALDI-TOF mass spectrometry. The isolates were collected consecutively. One isolate was generally for one patient. If there were multiple isolates of the same species, the first isolate was used. With the identification of the *S. aureus* from the colonies, oxacillin and clindamycin susceptibility tests are applied to label the two susceptibilities to the colonies. The technique and reagents are originated from the cefoxitin disk (Clinical and Laboratory Standards Institute guideline)^1^ for non-sterile specimens. For the sterile specimens including B specimens, the broth microdilution method is used as the resistance test.

Our cohorts were analyzed under MALDI-TOF MS (Microflex LT MALDI-TOF System, Bruker Daltonik GmbH). The operation requirement and processes were run under the manufacturer’s guidance. Each step is as follows. (1) prepare a MALDI steel target plate, smear the colonies after culture with a thin film adding formic acid (1 μl, 70%), and get dried at 25°C, (2) prepare the matrix solution based on the guidance and kit (1% α-cyano-4-hydroxycinnamic acid in 50% acetonitrile containing 2.5% trifluoroacetic acid), (3) add the matrix solution to the film and get dried under room temperature, and (4) microflex LT MALDI-TOF analyzer was operated to analyze the samples (linear ionization mode; accelerating voltage, 20 kV; nitrogen laser frequency: 60 Hz; 240 laser shots). In the end, we generated the raw MALDI-TOF data, whose *m*/*z* ratio ranged from 2,000 to 20,000 Da.

### MALDI-TOF Data Preprocessing and Pseudo-Ion Peak Intensity Matrix Generation

In the part of raw data preprocessing, three techniques were used to treat the data by order. An external calibration (Bruker Daltonics Bacterial Test Standard) was applied as the first step. Later, peak smoothing was performed using the Savitzky–Golay filter, and baseline correction was performed using the Top-hat filter. Peaks with a signal-to-noise ratio were set larger or equal to 2 for further analysis.

The preprocessed data based on the raw data consist of two categories of the variable for each sample: *m*/*z* and peak intensity. For the further preprocess, first, filter the unqualified mass spectra that the number of peaks is lower than 100 or larger than 200. Subsequently, by considering the sparsity of MS data when the *m*/*z* ratio is larger than 8,000 Da and signal regarding phenol-soluble modulin (PSM)-mec was studied earlier, which is a peptide with 2,415 *m*/*z* encoded by resistance gene, mecA (Josten et al., 2014), the MS data are extracted for each sample based on the range of *m*/*z* from 2,000 to 8,000 Da. Meanwhile, to minimize the impact of peak shift caused by different fragmentation results due to the initial point, the window size of 20 Da is considered to modify the data and transfer the *m*/*z* ratio into pseudo-ions. Specifically, the first pseudo-ion includes the intensity for the *m*/*z* ratio ranges from 2,000 to 2,010 Da as same as the last pseudo-ion. Other pseudo-ions between them stand for an interval lasting for 20 Da. In the end, a total of 301 pseudo-ions are generated, and the intensity corresponding to a pseudo-ion is the intensity sum within the interval. The intensity of the *i*th (*i* = 1,…,301) pseudo-ion for one sample can be calculated as follows:


intensity=′(i)∑j=1interval(i)intensity(j)

where in one sample, intensity′(i) stands for the intensity corresponding to *i*th pseudo-ion. Interval (*i*) refers to the *m*/*z* ratios within the interval, and *intensity*_(*j*)_ is the intensity for a specific *m*/*z* ratio.

### Multi-Label Classification

Multi-label learning studies the problem where each example is represented by a single instance while associated with a set of labels simultaneously (Zhang and Zhou, 2014). In this study, the pseudo-ion-intensity data are the observation data, and the results of the susceptibility test for oxacillin and clindamycin are assigned as the label data. All the multi-label learning algorithms are from scikit-multilearn 0.2.0 (Szymański and Kajdanowicz, 2017), a library for multi-label classification built on top of the scikit-learn ecosystem, using Python 3.68.

### Binary Relevance

Binary relevance is the most intuitive idea to deal with multi-label prediction. It treats the multi-label separately by considering multiple independent binary classifications for each label instead of viewing it as a group of labels. Like in this study, for the binary relevance, it needs to train two models, and the output is the union of two separated predictions.

### Classifier Chain

Classifier chain is the improved transformer of binary relevance by the construction of a Bayesian conditioned chain. Similar to the binary relevance, the classifier chain treats each label as a separated classier but not independent. Although the first classier is only trained using the input data (observation), the classifiers after are trained on the input space and all previous classifiers in the chain based on the Bayesian chain rule by order.

### Lowest Power Set

Unlike the previous two methods, the lowest power set is to transform a multi-label problem into a multi-class problem. Like this study, for 2 labels totally, it will eventually transform into a 4-class classifier.

### Quality Measures for Multi-Label Model

Two measurements are applied to evaluate the multi-label model in this study. The first measures are the hamming loss, which stands for the proportion of the incorrect prediction for all labels among the whole samples. The other measurement is the accuracy score. It means the fraction of samples for those prediction sets that exactly match the real label sets.

### Logistic Regression

Inside the multi-label algorithm, the classifier needs to be defined. Logistic regression is a common classifier to predict the resistance in the biological field (Moradigaravand et al., 2018). In this study, the discovery samples, the Linkou cohort, are used in the model training, while the Kaohsiung cohort is responsible for the independent test. The logistic regression (LR) model is realized using the Python package sklearn. Grid search is applied for the parameter tuning based on the criteria of the area under the receiver operating characteristic (ROC) curve by the adjustment of parameters including the penalty, *C*-value, and solver. Each model during the tuning is evaluated by the 5-fold cross-validation. For the tuned model, using L1 normalization as the penalty, 1 for the *C*-value, and liblinear, a library for large linear classification, as the solver is the tuned parameters. The area under the curve (AUC) will be applied to evaluate the training model in the replication cohort. The model training and parameter tuning are achieved in the Python package, scikit learn. The presentation of the ROC curve is generated from the Python package, Matplotlib.

### XGBoost

XGBoost is a scalable machine learning system for the tree boost, offering parallel tree boosting (Chen and Guestrin, 2016). Choosing XGBoost as the classifier in the multi-label model, such as the LR above, the Linkou cohort is treated as the training data, and the Kaohsiung cohort is used for the independent test by orders. Package xgboost from Python is applied to realized XGBoost model. Parameters shown in Table 3 were tuned through grid search. The result evaluation of the model is the same procedure as the LR.

**TABLE 3:** Parameters tuned for XGBoost under multi-label learning.

Parameter	Function	Tuned result
max_depth	Maximum depth of a tree	3
min_child_weight	Minimum sum of weight for a child	1
Gamma	Minimum loss requirement for node partition	0
subsample	Subsample ratio within the training samples	0.6
colsample_bytree	Subsample ratio of columns when constructing each tree	0.6

### Permutation Importance

Permutation importance is a technique used to generate the feature importance for the trained model. It is defined as the decrease of significance *P*-values for each feature when the value is randomly shuffled (Altmann et al., 2010).

## Results

### Performance of Multi-Label Prediction Learning Using Logistic Regression and XGBoost

To realize the goal of serial antibiotic resistances prediction, the study adopted three multi-learning ensembles provided by scikit-multilearn 0.2.0, including BinaryRelevance, ClassifierChain, and Lowest Power set. For each ensemble, the study applied LR and XGBoost as the classifier, respectively. Based on the ensembles and classifiers, the prediction model could provide prediction to the susceptibilities of oxacillin and clindamycin within one-step training among the Linkou cohort for each sample. The primary model evaluation among the Kaohsiung cohort is shown in Table 4.

**TABLE 4:** Model evaluation in multi-label ensembles using LR and XGBoost correspondingly.

Criteria\Ensembles	BinaryRelevance LR (XGBoost)	ClassifierChain LR (XGBoost)	Lowest Power set LR (XGBoost)
Hamming loss	0.2023 (**0.1622**)	0.2015 (**0.1628**)	0.2044 (**0.1524**)
Accuracy score	0.6863 (**0.7334**)	0.6885 (**0.7553**)	0.7119 (**0.7717**)
Jaccard score	0.6019 (**0.6677**)	0.6038 (**0.6676**)	0.6033 (**0.6839**)

*Hamming loss, accuracy score, and Jaccard score are used to evaluate the multi-label model primarily. Hamming loss refers to the average fraction of the wrong prediction of each sublabel. The accuracy score is based on the accuracy of the serial label prediction. Jaccard score measures the proportion of prediction for a sample to its true label. Bold values refer to better performance based on each criterion.*

From the evaluation criteria shown in Table 4, when applying XGBoost as the classifier in all of three multi-label ensembles, the performance in serial label prediction (Accuracy score) or partial label within the prediction (Hamming loss and Jaccard score) both indicated an improved model than using LR as the classifier. Based on the accuracy score, approximately 6% of improvement using XGBoost could be observed from 0.69 on average to 0.75 on average, which presents a refinement that exists in multi-label prediction for antibiotic susceptibility.

Besides the evaluation for the multi-label prediction, analysis that was related to partial or single susceptibility is conducted by dividing the serial label prediction for each sample into susceptibility prediction for oxacillin and clindamycin correspondingly. Herein, the ensemble Lowest Power set with LR and XGBoost is adopted to evaluate the partial performance for its best serial performance in Table 4. The evaluation information including precision and recall in each class and total accuracy among discovery and replication cohort is presented in Table 5. Both from the discovery and replication cohort, the ensemble that is applied with XGBoost shows more reliable performance than the ensemble that is applied with LR in oxacillin and clindamycin. The discovery accuracy gets increased from 0.83 (oxacillin) and 0.82 (clindamycin) to 0.89 and 0.88. The replication accuracy gets increased from 0.79 (oxacillin) and 0.80 (clindamycin) to 0.84 and 0.83.

**TABLE 5:** Evaluation of partial susceptibility prediction in discovery and replication cohort.

		Precision	Recall		Precision	Recall
Discovery	OSSA	0.81 (**0.88**)	0.85 (**0.90**)	CSSA	0.82 (**0.87**)	0.88 (**0.93**)
Linkou	ORSA	0.86 (**0.91**)	0.81 (**0.89**)	CRSA	0.82 (**0.89**)	0.75 (**0.82**)
Cohort	Accuracy		0.83 (**0.89**)			0.82 (**0.88**)
Replication	OSSA	0.78 (**0.82**)	0.83 (**0.88**)	CSSA	0.83 (**0.84**)	0.86 (**0.90**)
Kaohsiung	ORSA	0.80 (**0.86**)	0.74 (**0.79**)	CRSA	0.75 (**0.82**)	0.71 (**0.72**)
Cohort	Accuracy		0.79 (**0.84**)			0.80 (**0.83**)

*The data outside the bracket are generated by the Lowest Power set ensemble with logistic regression. The data within the bracket are generated by the Lowest Power set ensemble with XGBoost. Bold values refer to better performance based on each criterion.*

**FIGURE 3:**
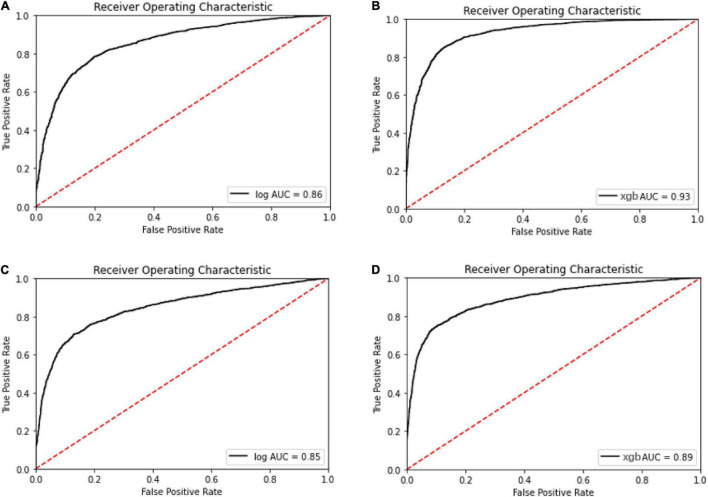
Receiver operating characteristic curve for the oxacillin and clindamycin susceptibility prediction under replication cohort in multi-label learning model, respectively. The area under the curve (AUC) is noted in the curve. **(A)** Receiver operating characteristic (ROC) for the oxacillin susceptibility prediction in replication cohort using Lowest Power set with logistic regression (LR); **(B)** ROC for the oxacillin susceptibility prediction in replication cohort using Lowest Power set with XGBoost; **(C)** ROC for the clindamycin susceptibility prediction in replication cohort using Lowest Power set with LR; and **(D)** ROC for the clindamycin susceptibility prediction in replication cohort using Lowest Power set with XGBoost.

Meanwhile, with the consideration of specificity and sensitivity of prediction models, the area under the ROC curve was used to measure the model performance. The AUC of oxacillin and clindamycin prediction using LR or XGBoost as the classifier in the multi-label learning model is shown in Figure 3. The AUC for oxacillin susceptibility prediction under XGBoost is 0.93, while it is only 0.86 for the model applying LR. The performance for oxacillin resistance prediction got improved compared with the AUC of 0.80 from DRIAMS (Weis et al., 2022) as well. Meanwhile, the AUC of clindamycin susceptibility prediction gets increased from 0.85 to 0.89 by turning LR into XGBoost.

**FIGURE 4:**
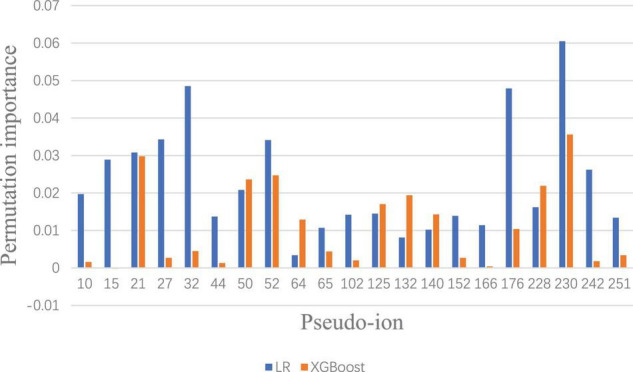
Significant permutation importance comparison between LR and XGBoost under Lowest Power set ensemble.

To visualize and identify the performance improvement, permutation importance of features under Lowest Power set using LR and XGBoost is conducted, respectively. After calculating the permutation importance in each model, each feature is assigned with an importance value ranging from 1 to −1, which indicates the feature contribution to the model performance. The whole permutation importance is shown in the Supplementary Material. To analyze the main feature importance between LR and XGBoost under Lowest Power set, features that contribute higher than 0.01 permutation importance in either LR or XGBoost model are extracted for comparison (Figure 4). Based on permutation importance comparison, the multi-label prediction model using Lowest Power set with LR mainly focuses on pseudo-ion with low and high *m*/*z*-ratio-relatively-in-the-MALDI-TOF-data-(pseudo-ions 10, 15, 27, 32, 44, 176, 230, and 242). For instance, the multi-label model using LR assigns high permutation importance to pseudo ion 15, which stands for protein fragments from 2,310 to 2,330 *m*/*z*, and pseudo ion 242, which stands for protein fragments from 6,850 to 6,870 *m*/*z*. Although the multi-label prediction model using XGBoost shown in Figure 4 indicates some shared important features, such as pseudo ions 21 and 230, it presents a focus on the medial features, such as pseudo ions 64 and 132. On the whole, the over-refinement between applying LR or XGBoost as the classier in the multi-label prediction model reflects on the relief of permutation importance in the protein fragments with low or high *m*/*z* ratio and a new focus on the medial pseudo ions.

### Performance of Multi-Label Prediction Learning and Single Label Prediction Learning

With the hypothesis that whether the model is trained under a single label or multi labels has an influence on the prediction performance, this study constructs two models for oxacillin susceptibility and clindamycin susceptibility, respectively, and separately using XGBoost. Precisely, it refers to using only one susceptibility label from the discovery cohort to train the XGBoost model for the prediction in the replication cohort. During the model training process, RandomOverSampler is adopted to balance the class size from the Python package, Imbalanced-learn (Lemaître et al., 2017). The multi-label prediction model, in this study, still applied XGBoost in the Lowest Power set ensemble.

**FIGURE 5:**
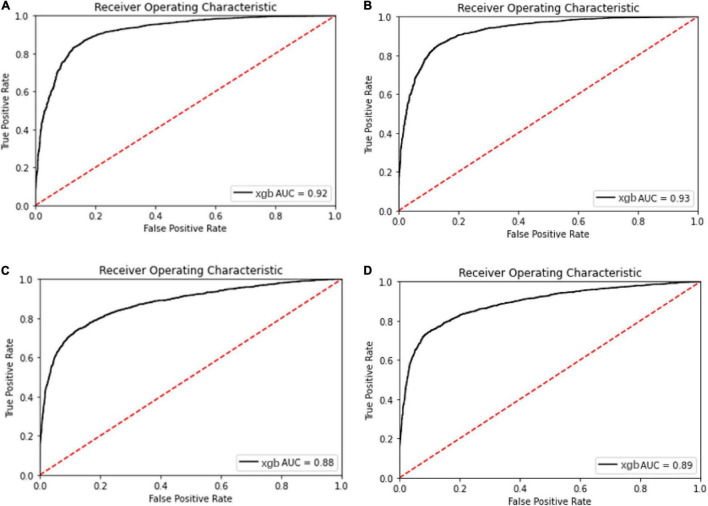
Receiver operating characteristic curve for the oxacillin and clindamycin susceptibility prediction under replication cohort in multi-label learning model, respectively. The AUC is noted in the curve. **(A)** ROC for the oxacillin susceptibility prediction in replication cohort using XGBoost; **(B)** ROC for the oxacillin susceptibility prediction in replication cohort using Lowest Power set with XGBoost; **(C)** ROC for the clindamycin susceptibility prediction in replication cohort using XGBoost; and **(D)** ROC for the clindamycin susceptibility prediction in replication cohort using L Lowest Power set with XGBoost.

The ROC and AUC for performance comparison among the multi-label prediction model and single label prediction model are shown in Figure 5. Initially, considering due to the multi-label learning, the boundary conditions or hyperplanes for the model may need to be relaxed relatively compared with the single label prediction. However, the model performance using XGBoost as the classier in the multi-label prediction model actually presents approaches to the single susceptibility prediction model and even better to the single one.

**FIGURE 6:**
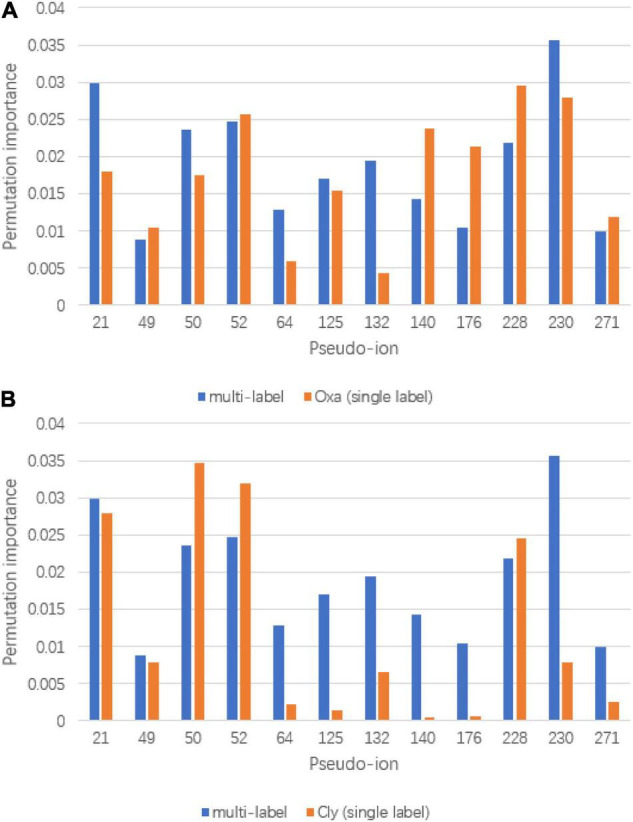
Significant permutation importance comparison between multi-label learning model and single label learning model. **(A)** Permutation importance comparison for oxacillin susceptibility prediction and **(B)** permutation importance comparison for clindamycin susceptibility prediction.

To visualize the refinement reflection, permutation importance provides insights to model construction. The full permutation importance of pseudo ion is attached to the Supplementary Document. The pseudo ions with permutation importance larger than 0.01 were used for comparison (Figure 6). For the oxacillin susceptibility prediction, within the multi-label prediction model, it shows a diverse focus among pseudo ions with different *m*/*z* ratios. For instance, the model assigned more significant permutation importance to pseudo ion 21 with its *m*/*z* ratio from 2,410 to 2,430, ion 50 with its *m*/*z* ratio from 3,010 to 3,030, ion 64 with its *m*/*z* ratio from 3,290 to 3,310, ion 132 with its *m*/*z* ratio from 4,650 to 4,670 and ion 230 with its *m*/*z* ratio from 6,610 to 6,630. In terms of clindamycin susceptibility prediction, the multi-label prediction model shows a consensus that it majorly focuses on pseudo ion with the *m*/*z* ratio from 3,000 to 4,000, determining higher permutation importance on pseudo ions 64, 125, 132, 140, and 176 than the single label model. Besides, compared with the single label prediction model for oxacillin and clindamycin susceptibility together, the multi-label learning model addresses pseudo ions 21, 64, 125, 132, and 230.

## Discussion and Conclusion

To deal with potential consideration for the efficiency and accuracy of detection, efforts during the whole experiment are conducted for the realization of a practical model. First, in our study, five kinds of specimens with oxacillin and clindamycin susceptibility labels were used to convince the model. In the future, more kinds of specimens could be used to strengthen our model. Second, the large size of the discovery cohort and the offer of replication cohort largely support the machine learning model. The size of 26,852 for the discovery cohort from 2013 to 2019 from the Linkou, Taiwan area, is fundamental to train the model solidly. Meanwhile, the replication cohort with 4,963 samples from Kaohsiung served as the validation set. Third, instead of constructing a machine learning model for only one antibiotic susceptibility, our study uses the Lowest Power set ensemble and applies XGBoost as the classifier to build up a multi-label prediction model, which could predict the susceptibilities of the oxacillin and clindamycin at the same time with only one-step training. From the model performance, the multi-label model combined with XGBoost shows better performance (AUC and accuracy) than choosing LR as the classifier, which is commonly used for susceptibility prediction in previous studies (Moradigaravand et al., 2018; Wang et al., 2020). In terms of the output type, the multi-label prediction model performs better than the single label model with only one training process. Furthermore, feature importance was used to analyze the improvement between models, and several potential biological insights were generated.

Based on the prediction performances between single label prediction model and multi-label prediction model and permutation importance results, feature contribution analysis was conducted. For the oxacillin susceptibility prediction, the dominant importance increase of pseudo ions 21 and 50, referring to 2,410–2,430 Da and 2,990–3,010 Da, respectively, matches with the research of Josten et al. (2014). Their study regarded the fragment with an *m*/*z* ratio of 2,413 Da as a marker for the presence of phenol-soluble modulin (PSM)-mec, which is a small excreted peptide encoded by the mec gene. Besides, the PSM-mec is excreted by agr-positive strains, where it presents with the delta-toxin with an *m*/*z* ratio of 3,007 Da. For the clindamycin susceptibility prediction, fragment around pseudo ion 64, the *m*/*z* ratio of 3,270 Da–3,290 Da is present to show the expression of Cfr. Gene Cfr induces the resistance to clindamycin. Beyond pseudo ion 64, ions 50 and 52 could be potential entry for biological insight analysis for their high permutation importance on clindamycin susceptibility prediction. The synergy importance increase occurred on pseudo ions 64, 132, and 230, covering *m/z* of 3,270–3,290 Da, 4,630–4,650 Da, and 6,590–6,610 Da. The hypothesis of synergy effect between the susceptibility of oxacillin and clindamycin or even among multidrug resistance could likely be tested by considering the three ions above.

There are several limitations and restrictions in our study. First, the discovery and replication cohorts are actually based on the local part in the Taiwan area, and the model needs more samples worldwide to become a practical susceptibility prediction model at a global level. Second, in our study, we only possessed and considered the susceptibilities of oxacillin and clindamycin, the simplest case of the multi-label prediction. There is a simple correlation analysis between two susceptibilities. In the future, during the sample recruitment, information about more than three kinds of susceptibilities could be tested and collected to realize more complex serial label predictions. Meanwhile, the statistical methods for isolate selection for MALDI-TOF need to be improved. Previous study has concluded that single isolate selection in MALDI-TOF may generate biased results if missed to identify the diversity among isolates (Pinar-Méndez et al., 2021). Considering the variation among isolates, the MALDI-TOF result from one isolate for each patient is not representative enough as the input data for susceptibility prediction. Optimized statistical methods are needed, such as multi-isolate selection or MS data integration from multi isolates, which could be conducted in the future study for a comprehensive prediction model. In addition, future studies can adopt a novel ensemble method that considers the relation among labels or susceptibilities instead of Lowest Power set in this study for better serial prediction performance. A platform or database combining resistance information, such as prediction or tendency with large sample size and diverse drug susceptibilities, could be continuous for future study. During the permutation importance analysis, some pseudo ions were pointed out to respond for the model refinement. These ions could be considered as the potential biomarker or functional segments and needed to analyze in the laboratory. Although the AUC for oxacillin and clindamycin susceptibility prediction indicates good performances, the accuracy for serial susceptibility prediction still does not satisfy the clinical requirements. However, our model presents the possibility of a proteomic-based model with a machine learning algorithm for rapid serial susceptibility prediction.

To summarize our study, we successfully constructed a multi-label prediction model applying XGBoost in Lowest Power set for oxacillin and clindamycin susceptibilities based on the MALDI-TOF MS data with the output of serial labels. Multidrug resistance is a threat to disturb treatment effects and usually tested by AST, which is limited by the labor and facility resource. Under large-scale size in the discovery cohort and replication cohort, our model could realize serial susceptibility prediction solidly, which ideally help patients and doctor with clinical guidance and insights to the antibiotic usage efficiently and accurately. In a nutshell, combing MALDI-TOF MS and machine learning algorithm will widely spread a proteomic-based antibiotic susceptibility test clinically taking advantage of speed and accuracy and saving the resources that originally are consumed for the costing and inefficient AST.

## Data Availability Statement

The original contributions presented in the study are included in the article/Supplementary Material, further inquiries can be directed to the corresponding author/s.

## Author Contributions

JZ, ZW, H-YW, and T-YL conceived the project, designed and conducted the analyses, interpreted the results, wrote the manuscript, and are listed in random order. H-YW collected the clinical samples and executed the microbiology experiments. JZ and ZW conducted the analyses and wrote the manuscript. JZ assisted with the machine learning analysis and visualization. C-RC and J-TH assisted with manuscript revision. J-JL and T-YL supervised the study. All authors have read and approved the manuscript.

## Conflict of Interest

The authors declare that the research was conducted in the absence of any commercial or financial relationships that could be construed as a potential conflict of interest.

## Publisher’s Note

All claims expressed in this article are solely those of the authors and do not necessarily represent those of their affiliated organizations, or those of the publisher, the editors and the reviewers. Any product that may be evaluated in this article, or claim that may be made by its manufacturer, is not guaranteed or endorsed by the publisher.

## Footnotes

1
www.clsi.org
